# Is it Possible to Have Coexisting Exogenous and Endogenous Cushing's Syndrome?

**DOI:** 10.7759/cureus.109011

**Published:** 2026-05-17

**Authors:** Sana Rafi, Nada El Idrissi Dfali, Ghizlane Elmghari, Nawal El Ansari

**Affiliations:** 1 Endocrinology and Metabolic Diseases, Hopital Errazi, Centre Hospitalier Universitaire (CHU) Mohamed VI de Marrakech, Marrakech, MAR

**Keywords:** coexisting, cushing’s disease, diagnosis, exogenous cushing’s syndrome, serum cortisol test

## Abstract

The coexistence of exogenous and endogenous Cushing’s syndrome is exceptionally rare. Here, we report the case of a 40-year-old female patient who had been self-medicating with dexamethasone for three years and who presented with asthenia and abdominal pain ten days after discontinuing corticosteroid therapy. The initial clinical suspicion was adrenal insufficiency following corticosteroid withdrawal; however, the patient’s 8 a.m. serum cortisol level was elevated at 46 µg/dL. The possibility of concomitant endogenous Cushing’s syndrome was therefore considered. Hormonal investigations confirmed adrenocorticotropic hormone (ACTH)-dependent Cushing’s syndrome, and magnetic resonance imaging of the pituitary region revealed a pituitary macroadenoma measuring 14 × 15 × 12 mm. The patient underwent transsphenoidal surgery with an uneventful postoperative course. Hormonal evaluation should be considered in patients receiving prolonged corticosteroid therapy when clinical manifestations persist after corticosteroid withdrawal, particularly in the presence of persistent hypercortisolism or absence of expected hypothalamic-pituitary-adrenal axis suppression.

## Introduction

Cushing’s syndrome (CS) results from prolonged exposure to glucocorticoids. Exogenous CS is the most common, given the widespread use of corticosteroid therapy; it affects 2-3% of the general population, but there are no data on the incidence of exogenous CS [1,2]. As for endogenous CS, it is rare, with an estimated incidence of 1.8 to 3.2 cases per million people per year [1,3].

Endogenous CS may be adrenocorticotropic hormone (ACTH)-dependent, most commonly caused by pituitary corticotroph adenomas (Cushing’s disease) or ectopic ACTH secretion, or ACTH-independent, mainly related to adrenal adenomas, adrenal carcinomas, or adrenal hyperplasia [1-3]. In contrast, exogenous CS results from prolonged exposure to corticosteroids in oral, injectable, topical, or inhaled forms [1,2]. Chronic corticosteroid exposure usually suppresses the hypothalamic-pituitary-adrenal axis [2].

The association or coexistence of exogenous and endogenous CS is very rare, and only a few cases have been reported in the literature [4-6].

Here, we report the case of a patient presenting with exogenous and endogenous CS, whose diagnosis was very challenging.

## Case presentation

A 40-year-old female patient, hypertensive for three months and treated with 5 mg of amlodipine, with newly diagnosed untreated diabetes mellitus, presented to the emergency department with severe asthenia, abdominal pain, and vomiting. The medical history revealed prolonged self-medication with dexamethasone at a dose of 1 mg/day for three years in order to gain weight. The patient reported having ceased this corticosteroid therapy 10 days prior to presentation.

**Figure 1:**
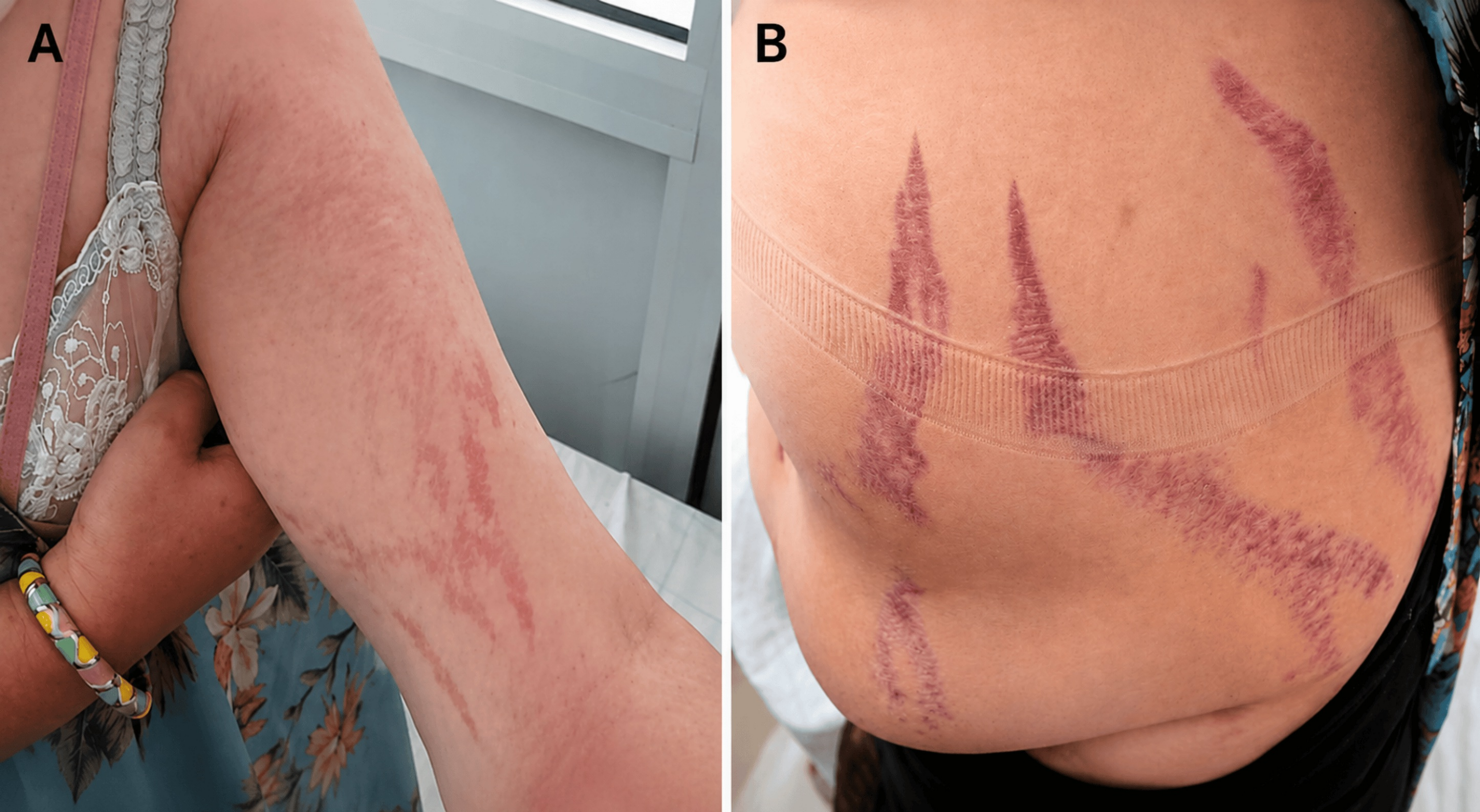
Purple stretch marks on the abdomen and at the base of the upper limb. (A) Purple striae at the base of the upper limb; (B) purple abdominal striae.

Physical examination revealed a blood pressure of 125/80 mm Hg and a capillary blood glucose level of 1.80 g/L (reference range: 0.70-1.10 g/L). The patient had a moon face, a buffalo hump, centripetal obesity contrasting with thin limbs, and purple striae on the abdomen and at the base of the upper limbs (Figure 1).

An initial diagnosis of suspected adrenal insufficiency following corticosteroid withdrawal was considered. An 8 a.m. serum cortisol level was ordered, together with a comprehensive biochemical evaluation. Treatment was initiated, and the patient was admitted to our department.

The 8 a.m. serum cortisol level was 46 µg/dL (reference range: 3.7-19.4 µg/dL), the serum sodium level was 142 mmol/L (reference range: 135-145 mmol/L), and hypokalemia was noted at 2.7 mmol/L (reference range: 3.5-4.5 mmol/L). These biochemical findings were inconsistent with the expected hypothalamic-pituitary-adrenal axis suppression observed after prolonged corticosteroid exposure. The possibility of concomitant endogenous CS was therefore raised.

**Figure 2:**
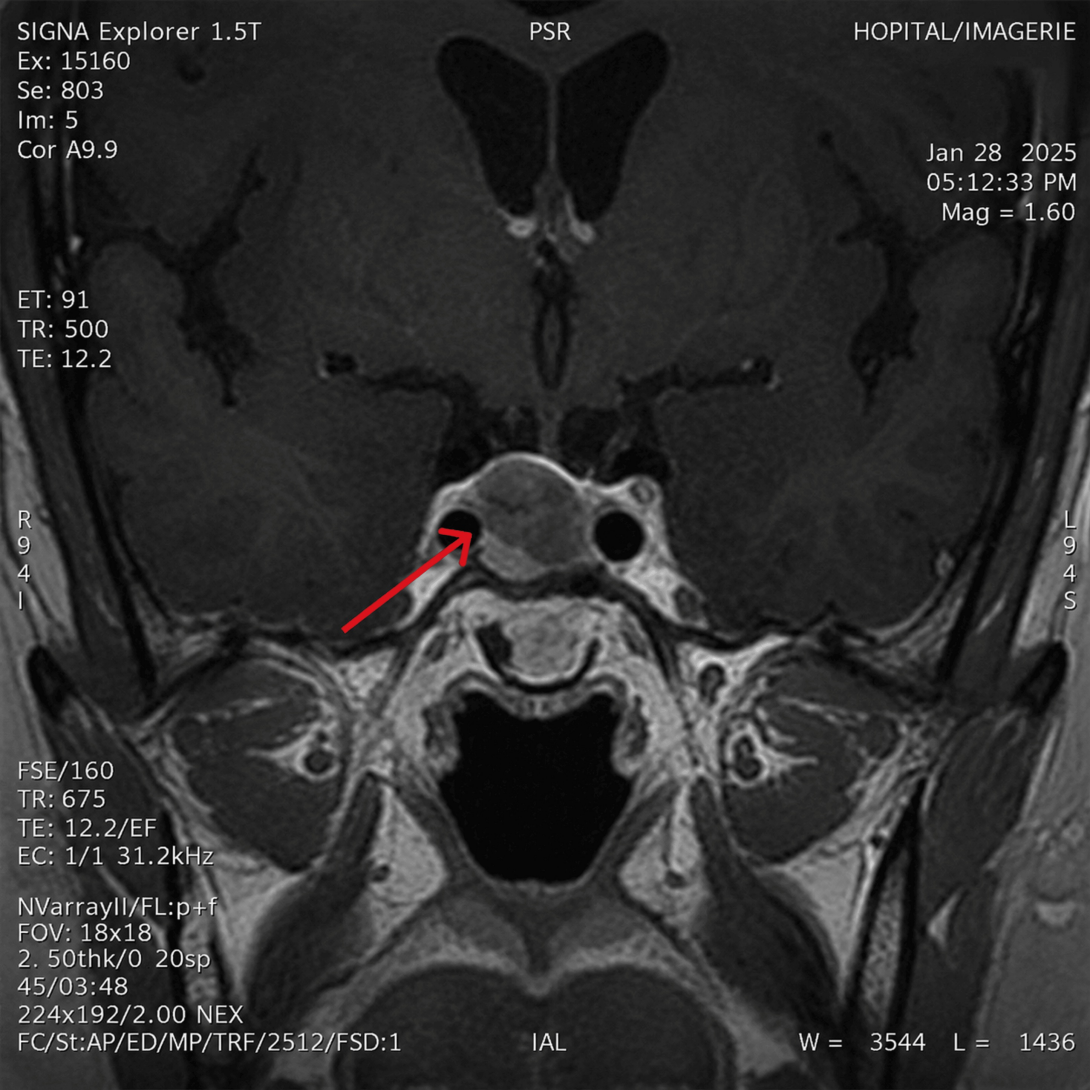
Magnetic resonance imaging of the pituitary region, coronal section, necrotic-hemorrhagic sella turcica measuring 14 x 15 x 12 mm.

Biochemical investigations confirmed the diagnosis of ACTH-dependent CS (Table 1), and magnetic resonance imaging (MRI) of the pituitary region revealed a sellar lesion measuring 14 × 15 × 12 mm, consistent with a pituitary macroadenoma with necrotic-hemorrhagic changes (Figure 2).

**Table 1:** Hormonal evaluation of endogenous ACTH-dependent Cushing’s syndrome. ACTH, adrenocorticotropic hormone.

Parameters	Results	Normal values
Minimal response to dexamethasone	21.3 µg/dl	<1.8 µg/dl
24-hour urinary free cortisol	257.46 µg/24h	4.3-176
ACTH	94 pg/ml	4.7-48.8

A diagnosis of Cushing’s disease was established, and the patient underwent transsphenoidal surgery. The postoperative course was uneventful; however, the patient subsequently developed postoperative adrenocortical insufficiency with an 8 a.m. serum cortisol level of 2 µg/dL. Histopathological examination revealed morphological and immunohistochemical features consistent with a pituitary neuroendocrine tumor expressing anti-ACTH antibodies, with a Ki-67 proliferation index of 2%.

## Discussion

CS is rare [1], and the most common cause is exogenous CS, secondary to prolonged corticosteroid therapy, generally persisting for more than three months [1,7]. When the origin is endogenous, the most common cause is ACTH-dependent CS in approximately 80% of patients, with Cushing’s disease remaining the most frequently identified etiology [1,3].

The coexistence of exogenous and endogenous CS is very rare [4,6], and this atypical association may lead to delayed diagnosis and management [1,3,6]. In the present case, the diagnostic challenge was further complicated by prolonged self-medication with corticosteroids, and the patient only sought medical attention after discontinuing corticosteroid therapy.

The diagnosis of this association is based on the absence of expected hypothalamic-pituitary-adrenal axis suppression after prolonged corticosteroid exposure [4-6]. Coexisting endogenous CS should be suspected in patients receiving long-term corticosteroid therapy when clinical manifestations persist after corticosteroid withdrawal, particularly in the presence of persistent hypercortisolism, hypertension, hypokalemia, or absence of expected hypothalamic-pituitary-adrenal axis suppression.

The clinical presentation may overlap between exogenous and endogenous CS; however, while striae are reportedly more pronounced in exogenous CS, acne and hirsutism are more commonly associated with endogenous CS [6,8,9]. Hypertension and hypokalemia are also more frequently reported in endogenous CS, as observed in the present case [6,9].

Only a few cases have been described in the literature thus far. The first involved a 23-year-old female patient treated for juvenile idiopathic arthritis with long-term corticosteroid therapy. The patient presented with signs of exogenous hypercortisolism, including growth retardation and severe bone loss. Evaluation of the hypothalamic-pituitary-adrenal axis revealed ACTH-independent CS, and imaging demonstrated adrenal micronodular hyperplasia [4].

The second case involved a 66-year-old female patient who developed clinical features of exogenous CS following one year of glucocorticoid intake in the form of medicinal plants. One year after corticosteroid discontinuation, she presented with a hypertensive crisis associated with persistent clinical and biochemical manifestations of hypercortisolism. Evaluation revealed ACTH-independent CS, and imaging demonstrated an adrenal adenoma [5].

Finally, the third case involved a 46-year-old male patient treated with corticosteroids for three years following post-coronavirus disease 2019 respiratory complications. The patient presented with clinical features initially consistent with exogenous CS, associated with hypertension and severe muscle weakness. Following a three-day interruption of corticosteroid therapy, testing revealed an elevated 8 a.m. serum cortisol level and biochemical findings consistent with ACTH-independent CS, with imaging revealing an adrenal adenoma [6].

These previously reported cases, together with the present case, confirm that the coexistence of exogenous and endogenous CS is possible and should be considered when the clinical presentation or biochemical findings are atypical after corticosteroid withdrawal.

## Conclusions

The coexistence of exogenous and endogenous CS is exceptionally rare and may represent a major diagnostic challenge. This report highlights the importance of maintaining a high index of suspicion for endogenous CS in patients receiving long-term corticosteroid therapy when the clinical course or biochemical findings are atypical. Persistent hypercortisolism, hypertension, hypokalemia, or the absence of suppression of the hypothalamic-pituitary-adrenal axis after corticosteroid discontinuation should prompt a comprehensive hormonal evaluation. Early recognition of this rare association is essential to avoid delayed diagnosis and ensure timely and appropriate management.
